# Identification of Rare *LRP5* Variants in a Cohort of Males with Impaired Bone Mass

**DOI:** 10.3390/ijms221910834

**Published:** 2021-10-07

**Authors:** Maria Santa Rocca, Giovanni Minervini, Andrea Di Nisio, Maurizio Merico, Maria Bueno Marinas, Luca De Toni, Kalliopi Pilichou, Andrea Garolla, Carlo Foresta, Alberto Ferlin

**Affiliations:** 1 Unit of Andrology and Reproductive Medicine, Department of Medicine, University Hospital Padua, 35128 Padua, Italy; mariasanta.rocca@aopd.veneto.it (M.S.R.); andrea.dinisio@unipd.it (A.D.N.); maurizio.merico@aopd.veneto.it (M.M.); luca.detoni@unipd.it (L.D.T.); andrea.garolla@unipd.it (A.G.); carlo.foresta@unipd.it (C.F.); 2 Department of Biomedical Sciences, University of Padova, 35121 Padova, Italy; 3 Department of Cardiac-Thoracic-Vascular Sciences and Public Health, University of Padua, 35121 Padua, Italy; maria.bueno.m@gmail.com (M.B.M.); kalliopi.pilichou@unipd.it (K.P.); 4 Unit of Endocrinology and Metabolism, Department of Clinical and Experimental Sciences, University of Brescia and ASST Spedali Civili Brescia, 25123 Brescia, Italy; alberto.ferlin@unipd.it

**Keywords:** impaired bone microstructure, BMD, computational study, NGS panel, LRP5

## Abstract

Osteoporosis is the most common bone disease characterized by reduced bone mass and increased bone fragility. Genetic contribution is one of the main causes of primary osteoporosis; therefore, both genders are affected by this skeletal disorder. Nonetheless, osteoporosis in men has received little attention, thus being underestimated and undertreated. The aim of this study was to identify novel genetic variants in a cohort of 128 males with idiopathic low bone mass using a next-generation sequencing (NGS) panel including genes whose mutations could result in reduced bone mineral density (BMD). Genetic analysis detected in eleven patients ten rare heterozygous variants within the *LRP5* gene, which were categorized as VUS (variant of uncertain significance), likely pathogenic and benign variants according to American College of Medical Genetics and Genomics (ACMG) guidelines. Protein structural and Bayesian analysis performed on identified *LRP5* variants pointed out p.R1036Q and p.R1135C as pathogenic, therefore suggesting the likely association of these two variants with the low bone mass phenotype. In conclusion, this study expands our understanding on the importance of a functional LRP5 protein in bone formation and highlights the necessity to sequence this gene in subjects with idiopathic low BMD.

## 1. Introduction

Osteoporosis is the most common skeletal disorder characterized by reduced bone mineral density (BMD), resulting in an increased risk of fragility fractures [1]. According to WHO criteria, osteoporosis is defined as a BMD of less than 2.5 SD (standard deviation) as compared to an average healthy population of the same age and gender.

Age, gender, and ethnicity are known unchangeable risk factors of this complex disorder; however, studies on monozygotic twins have shown that genetic factors account for 50–85% of cases of osteoporosis [2,3,4,5].

In addition to the monogenic form of osteoporosis due to rare mutations in well-known genes, genetic variants, referred to as polymorphisms or copy number variations, within or near genes encoding proteins involved in the molecular pathways underlying the maintenance of bone metabolism, could result in reduced BMD, leading to bone fragility.

Genome-Wide Association Studies (GWASs) have, indeed, identified genetic variants associated with an increased risk of fractures in genes encoding proteins that are involved in the metabolic balance of osteoblasts/osteoclasts [6,7].

Although osteoporosis affects both genders, it has been mostly studied in postmenopausal women due to hormonal imbalances as compared to men. This could explain why, for a long time, osteoporosis has been underestimated and undertreated in male patients [8].

The Low-Density Lipoprotein-Related Receptors 5 (LRP5) protein is a member of the LDL receptor superfamily and acts as co-receptor together with Frizzled for Wnt proteins to regulate the canonical Wnt/β-catenin signaling cascade leading to bone formation [9,10,11]. Therefore, mutations in the *LRP5* gene could alter normal Wnt/β-catenin signaling, causing altered bone formation. LRP5 is expressed in several tissues, including bone, where it is mainly expressed by bone-forming cells, such as osteoblasts.

Although homozygous loss-of-function and gain-of-function mutations in the *LRP5* gene (OMIM #603506) have been previously described as causative of osteoporosis pseudoglioma syndrome (OPGG) (MIM 259770) and high BMD syndrome (MIM 601884) [12,13], respectively, recent evidence has suggested that polymorphisms of this gene could also the cause of reduced bone mass and size [14,15,16].

*LRP5* maps to chromosome 11q12-13 and it has been frequently investigated in subjects with skeletal diseases as a candidate susceptibility factor for osteoporosis, as variants in this gene have reached genome-wide significance levels (*p* < 1.0 × 10^−21^) [17].

The main aim of this study was to identify novel genetic variants causative of idiopathic low bone mass, sequencing a cohort of 128 males with reduced bone mass using a NGS panel including genes associated with reduced BMD.

## 2. Results

### 2.1. Genetic Analysis

A total of 6558 single nucleotide changes were detected in target regions, whose 4444 were in the noncoding region. In the coding regions, we identified a total of 1372 synonymous and 742 nonsynonymous nucleotide variants, while nonsense and frameshift variants were not detected.

Of the detected nonsynonymous variants, 10 heterozygous missense variants had a minor allele frequency (MAF) lower than 1% and were localized within the *LRP5* gene. These rare missense variants were classified as VUS (variant of uncertain significance), likely pathogenic or benign according to ACMG criteria (Table 1).

No statistically significant difference in T and Z score between subjects with and without variants in *LRP5* was found (T_L_ *p* = 0.53 and T_TH_ *p* = 0.62; Z_L_ *p* = 0.7 and Z_TH_ *p* = 0.43, respectively).

Of 11 subjects with rare variants in *LRP5*, the patient with p.R1135C had a referred family history of osteoporosis. However, the unavailability of parental DNA did not allow us to determine the inheritance pattern of the identified variant.

### 2.2. Protein Structural Analysis

#### 2.2.1. LRP5 Sequence Conservation and Domain Analysis and Investigation of Variants

A search against the ClinVar database showed that almost all identified *LRP5* variants, except p.R1342P, are described as VUS. Notably, the p.R1036Q was previously linked to postmenopausal osteoporosis and OPPG; however, ClinVar marks this variant as having controversial interpretations. To better investigate their putative pathogenic role, we performed a sequence conservation analysis of LRP5. We found that most of the variants identified in this study affect not fully conserved regions (Supplementary Figure S1), apparently suggesting a neutral effect. Five variants, p.V99L, p.E341K, p.G333S, pT443M, p.R1036Q, and p.R1135C, map within different β-Propeller domains, while the p.G333S localizes in a linker region connecting the first two β-Propeller domains. A further four variants, p.R1342P, p.A1525V, p.A1537V, and p.S1585L, affect the cytoplasmic tails of LRP5. This region contains multiple PPSP motifs and, therefore, is thought to participate in the Wnt canonical signaling pathway [18].

#### 2.2.2. Self-Analysis of LRP5 Repeated Domains

**Figure 1:**
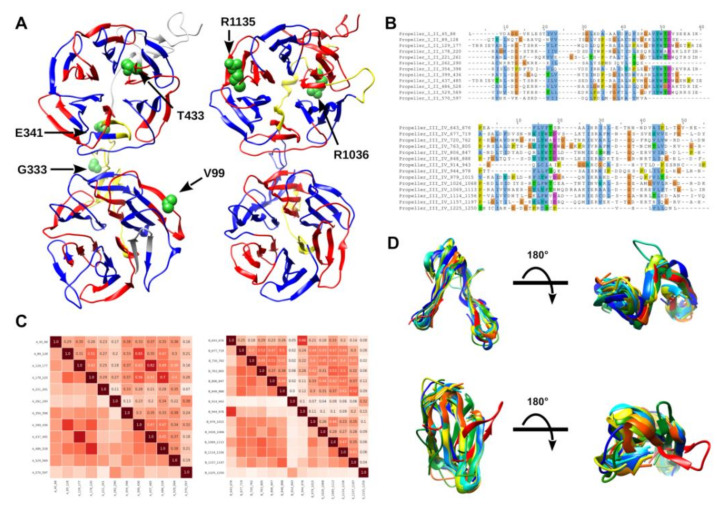
3D-model prediction of LRP5 repeats domains and self-analysis of repeated units. (**A**) Cartoon representation of β-Propeller domains I–IV with repeated units colored by localization. Residues found mutated are presented as green spheres. Sequence insertions are presented in yellow; (**B**) auto-alignment of β-Propeller domains highlighting the internal evolutive conservation of each unit; (**C**) heat-map showing the conservation score calculated by self-alignment of LRP5 protein sequence; (**D**) superimposition of structural units forming the β-Propeller domains I–IV.

The sequence-based analysis of repeat proteins is particularly challenging for standard bioinformatics analyses due to their repetitive and highly degenerated sequences [19]. The pathogenic interpretation of variants affecting repeated domains can be particularly difficult due to the scarce sequence conservation among ortholog sequences belonging to the same protein family. To address this issue, we adopted a self-analysis approach based on the self-alignment of β-Propeller domains to identify fully conserved positions putatively relevant for the folding of these domains. By using the SWISS-model webserver, we first generated two homology models, each covering a pair of β-Propeller domains (Figure 1), and used these 3D structures to perform self-analysis of the LRP5 repeated domains with RepeatsDB-lite [20]. The four main domains are correctly predicted to fold in distinct β-propellers; nevertheless, we observed notable differences in the conservation of repeat units. Indeed, propellers I and II are predicted to be constituted by the repetition of an “A-shaped” module, while propellers III and IV show a more conventional “extended β-sheet”-shaped unit. These findings suggest that these domains may present different folding stability, albeit sharing a similar 3D organization.

Considering these structural characteristics, we wondered whether these variants may promote a structural impairment of propellers. Variants p.V99L, p.E341K, and pT443M are predicted to not induce a relevant modification of protein structure. Based on our structural model, p.V99L resides within propeller I, localizing in a partially exposed region formed by two overlapping blades. Our 3D model predicts Val99 to form a van der Waals (vDW) interaction with Trp79, and its substitution with leucine should have a neutral effect. Variants p.G333S and p.E341K localize in a solvent-exposed linker region connecting propellers I and II, and both substitutions introduce polar residues. We predict these variants as neutral or to have a modest effect on the LRP5 structure. Unlike this, we predict p.T443M to have a stabilizing effect; indeed, the substitution of Thr433 in methionine introduces a further vDW interaction with Ile464, thus increasing contacts between residues from the last two blades of propeller II.

Finally, no structural investigation was conducted for p.R1342P, p.A1525V, p.A1537V, or p.S1585L, as we were unable to predict a reliable 3D structure of the LRP5 C-terminal tail. Prediction with MobiDB indeed [21] showed this region to be intrinsically disordered, thus lacking a fixed 3D-structure, suggesting that the pathogenic effect of these variant is not linked to the alteration of the LRP5 protein fold.

#### 2.2.3. Pathogenicity and Stability Predictions

To improve the reliability of our predictions, we decided to pair our structural analysis with pathogenicity and stability predictions. To this aim, we used a consensus approach based on four different in silico predictors. In general, variants were mostly classified as neutral or tolerated, while only the substitution p.S1585L was predicted as damaging by all predictors (Table 2).

Interestingly, this variant localizes between two PPSP motifs present on the LRP5 C-terminal tail. The PPSP is a reversible phosphorylation site motif shared by members of the LDL Receptor-Related Protein family, and it is known to be important in both mediating and regulating the interaction of these proteins with Wnt proteins [22,23]. Thus, mutations in this area may impair the correct phosphorylation of LRP5 and interfere with the normal Wnt signaling pathway. Interestingly, variants localizing within propeller IV, such as p.R1036Q and p.R1135C, were classified as damaging by three predictors, suggesting that these substitutions may induce a structural rearrangement of this domain. Similarly, the variant p.R1342P is mostly predicted as damaging. Prosite [24] predicted Arg1342 as the first residue of a motif involved in the formation of a disulfide bond between Cys1343 and Cys1361 (PROSITE-ProRule:PRU00124). Substitution with proline may impair disulfide bond formation and promote a local structural unfold.

#### 2.2.4. In Silico Classification of LRP5 VUS, Definition of a Prior Odds Ratio

Considering the scarcity of data for the pathogenic interpretation of LRP5 variants, we developed a Bayesian classificatory model using our results paired with experimental data from the literature. To classify the pathogenicity of each variant, we defined a prior odds ratio (OR) table containing the a priori risk probability for each mutation (Table S1, Supporting material). As previously proposed in [25,26], we assigned a prior OR of 19 (95:5) to LRP5 mutations described in the literature as pathogenic. A prior OR of 1.25 (5:4) was assigned to variants described in the dbSNP database as low frequency with no experimental data support the pathogenic effect, while a general prior OR of 1 (50:50) was assigned to the remaining variants. Multiple lines of evidence derived by different databases and computational tools were used to compile a prediction score for each variant and to assess their pathogenicity (detailed description in Method section).

#### 2.2.5. OR for Pathogenicity for In Silico Analysis

The pathogenicity risk derived by our model was presented using five different classes, namely Pathogenic, Likely Pathogenic, Uncertain, Likely Neutral, and Neutral, with each class associated with a decreasing value from 5 down to 1. Considering the computational nature of our assessment paired with a lack of functional validation, we decided to assign a high pathogenicity risk only to variants with a minimum agreement of 62.5% (agreement among pathogenicity descriptors > 5/8), i.e., class 5 and class 4. Similarly, to avoid any underestimation, we cautiously set the probability in favor of pathogenicity, assigning neutral and neutral-like (class 1 and class 2, respectively) to variants scoring below 25% (agreement among pathogenicity descriptors < 2/8). Variants presenting intermediated values were classified as uncertain. The resulting classification is shown in Table 3.

#### 2.2.6. Molecular Dynamics Simulation of p.R1036Q and p.R1135C Variants

**Figure 2:**
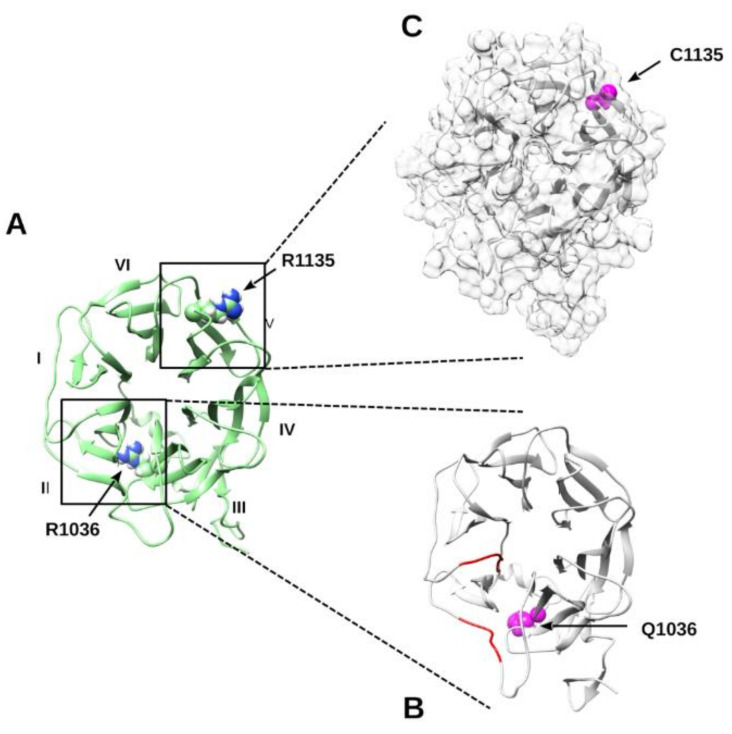
Molecular dynamics simulations of LRP5. (**A**) Cartoon representation of the wild-type β-Propeller IV after 250 ns of simulation. Roman numbers are for different blades forming the propeller. Variants are presented as spheres. (**B**) The same domain harboring the p.R1036Q substitution. Secondary structure elements lost after 250 ns of simulation are colored in red. (**C**) Transparent surface representation showing the solvent-accessible C1135.

We wondered whether the pathogenic effect predicted for both variants p.R1036Q and p.R1135C may rely on structural rearrangements of the LRP5 propeller IV. The molecular dynamics simulation showed p.R1036Q to induce a relevant structural unfold of the second blade forming propeller IV. This disruptive effect is due to impairment of an ionic interaction between Arg1036 and Asp985. The p.R1036Q mutant protein is indeed characterized by a loss of secondary structure in two fragments spanning residues 979–984 and 1011–1015, which became evident after 250 ns of simulation. The same regions remain stable without a relevant loss of secondary structure in the wild-type LRP5 protein under the same simulation conditions (Figure 2). These findings strongly suggest that p.R1036Q could impair the LRP5 biological function by promoting a partial unfold of the repeated unit IV. Unlike this, no evident unfold or structural rearrangements are predicted for the p.R1135C variant. Both WT and p.R1135C simulations show a similar dynamic behavior, suggesting that the pathogenic effect is not linked with LRP5 structural reorganization. Our simulations, however, show that the p.R1135C introduces a solvent-exposed cysteine residue (Figure 2). Considering the natural tendency of this specific amino acid to form a disulfuric bridge, we believe that this specific variant may form unwanted covalent protein–protein complexes, thus impairing the physiologic LRP5 functions.

## 3. Discussion

Osteoporosis is a polygenic disorder that affects about one in three women and one in five men over the age of 50 years [27].

Although it has been extensively studied in postmenopausal women, it is likely that this skeletal disorder may be underestimated and undertreated in male subjects, due also to the low number of male subjects undergoing bone density scanning [8].

As BMD and osteoporotic fractures are highly heritable [28], genetic tests should be recommended in individuals with a family history of fractures or with juvenile-onset primary osteoporosis, regardless of the gender.

Based on the growing body of evidence suggesting gene variants as a major risk for osteoporosis-related phenotypes [7,16,29], in this study, we sequenced 128 male subjects with idiopathic low bone mass in order to identify rare genetic variants that could be causative of the low BMD phenotype of our patients.

NGS analysis identified ten rare variants in the *LRP5* gene in eleven patients.

The *LRP5* gene maps to chromosome 11 and consists of 23 coding exons. It encodes a protein that functions as co-receptor that triggers the β-catenin pathway by binding Wnt proteins with the Frizzled-receptor. This molecular signaling is known to be involved in osteoblastic differentiation and proliferation [30]; therefore, the alteration of this mechanism could lead to an altered bone mass phenotype likely due to an impairment of bone microstructure.

**Figure 3:**
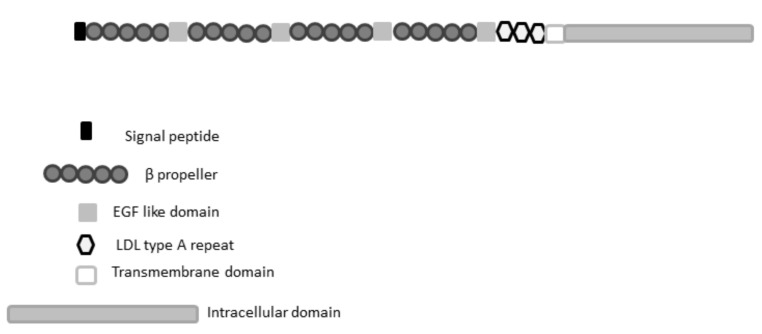
Schematic presentation of the protein structure and domain organization of LRP5.

The LRP5 protein consists of a large extracellular domain, four beta-propeller (YWTD) motifs and four epidermal growth factor (EGF)-like repeats, three LDLR type A domains, a transmembrane domain (TM), and a cytoplasmic domain (Figure 3).

The intracellular domain presents five PPPSP motifs, whose phosphorylation is essential to activate Wnt/β-catenin signaling.

Of the ten variants in *LRP5*, four were located in the second propeller domain, two in the fourth propeller domain, and the remaining six variants localize in the C-terminal tail (precisely, in the so-called LDLR type A repeats and PPPSP motif); however, only p.R1036Q and p.R1135C variants within the fourth propeller domain were predicted as pathogenic by both molecular dynamics simulations and Bayesian model.

The p.R1036Q has been described in other studies [31,32,33] that detected this variant in patients with OPPG [32] and juvenile osteoporosis [31,32] and observed a decreased *5-Htr1b* expression and the ability of LRP5 to modulate the Wnt-induced signaling pathway compared to wild-type, although functional results were not statistically significant [33]. The prediction of p.R1036Q as pathogenic by molecular dynamic results hence suggests that it could be the cause of low bone mass of the patient, and this hypothesis is corroborated from the observation of the same variant in patients with clinical signs. However, the more severe clinical phenotype reported in published cases could be due to biallelic p.R1036Q in these patients [32].

Although there are no functional studies or case reports describing the p.R1135C variant, it was predicted as pathogenic by structural protein analysis. It is located within the fourth beta propeller, and that with the third beta propeller forms the binding site of the Wnt3a ligand [34], whose signaling stimulates bone remodeling [35]. Therefore, the amino acid replacement could negatively impact on the ability of the protein to modulate this molecular pathway, for instance, participating in unphysiological protein–protein interactions mediated by the introduced solvent-exposed cysteine, resulting in reduced bone mass and risk of osteoporosis. Moreover, although our simulation does not predict p.R1135C as responsible of LRP5 local structural reorganization, we cannot exclude that it may impact on the LRP5 global fold. Indeed, the patient with the p.R1135C variant showed a severely reduced T and Z score and referred family history of osteoporosis, which are suggestive of a genetic bone mass trait. Therefore, it is essential to perform a future functional study of this variant in order to better define its role on bone mass.

Even though p.R1036Q and p.R1135C were predicted as putatively pathogenic by our computational investigation, patients harboring these variants share the osteoporosis phenotype comparable to patients with variants predicted as benign or likely pathogenic. Although this difference could likely depend on unidentified variants located in other genomic regions not covered by our gene panel, we cannot exclude a nonobvious impact of the benign variants on the phenotype. Indeed, in order to accurately attribute a clinical significance to each detected variant, future functional studies or animal models should be performed. Nevertheless, osteopenia/osteoporosis is clearly a multifactorial condition in which LRP5 mutations are one of the multiple possible causes.

Molecular dynamics simulations of variants in the C-terminal tail were not performed, due to the lack of a 3D structure model; however, they were all predicted as likely pathogenic by a Bayesian model. Of these variants, Crabbe et al. [36] described A1537 in a family whose members showed osteopenia/osteoporosis, even though Alanine residue was substituted by Threonine (A1537T). Despite the variant reported in the literature resulting in a different amino acid replacement, our patient and the proband described from Crabbe shared the same phenotype, namely low bone mass, therefore suggesting an important role of A1537 on protein structure.

Overall, this study contributes to an increase in the number of studies on male reduced bone mass and emphasizes the necessity to perform genetic screening in young and middle-aged men with idiopathic low bone mass in order to expand the understanding of the genetic causes leading to altered bone mass. Furthermore, the detection of variants in *LRP5* in approximately 8.6% (11/128) of analyzed subjects supports the importance of the LRP5 protein in the bone phenotype.

However, this study has a major limitation in the lack of a functional study. Indeed, although genetic analysis identified variants that could likely be associated with clinical phenotype, as supported by structural protein and Bayesian model analysis, functional assays are indispensable to better elucidate the role of identified variants.

In conclusion, this study confirmed by molecular dynamics analysis and a Bayesian model the likely pathological effects of some *LRP5* variants, thus supporting their role on low BMD phenotype, and, furthermore, highlighted the necessity to perform genetic analyses in subjects with idiopathic low bone mass in order to find new disease genes that may explain the impairment of bone microstructure.

## 4. Materials and Methods

### 4.1. Subjects

The study was approved by the Hospital Ethics Committee (no. 3389/U16/14), and each participant gave written informed consent.

A quantity of 223 male subjects of Caucasian ethnicity and Italian origin having a low BMD, defined as Z score and T score below −2.0 in subjects under and over 50 years, respectively, at the lumbar spine or at the proximal femur, associated or not with osteoporotic fractures, were referred to the clinic for management of low bone mass diagnosed by BMD tests. In all patients, careful medical history and physical examination were performed.

Exclusion criteria were: age > 60 years, malignant disease, medical disorders or medications interfering with bone metabolism, Paget disease, malabsorption, hypogonadism, hemochromatosis, hyperthyroidism, hypercortisolism, vitamin D deficiency, or mastocytosis.

The measurement of bone densitometry was performed by dual-energy X-ray absorptiometry using a Hologic QDR 4500 C densitometer (Hologic, Waltham, MA, USA) in the femoral neck (FN) and lumbar spine (L1 to L4), by the same technician, and a spine phantom was used before each examination.

Results were expressed as absolute BMD values (g/cm^2^), Z-scores, and T-scores obtained by a comparison with mean values of age- and young sex-matched persons, respectively, of the healthy population.

Of the total 223 enrolled patients, 128 subjects fulfilled the criteria for idiopathic altered bone mass.

Table 4 summarizes general characteristics of the patients.

### 4.2. DNA Isolation and Sequencing Analysis

Genomic DNA was extracted from peripheral blood leucocytes of subjects using the QIAamp DNA Blood Mini Kit, according to the manufacturer’s protocol (Qiagen Inc., Hilden, Germany). The quality of the DNA was determined using a NanoDrop-1000 (Thermo Fisher Scientific Inc., Waltham, MA, USA) and Qubit 2.0 fluorometer (Thermo Fisher Scientific Inc., Waltham, MA, USA). A Qubit dsDNA BR (broad range, 2 to 1000 ng) Assay Kit and Qubit dsDNA HS (high sensitivity, 0.2 to 100 ng) Assay Kit were used with a Qubit fluorometer according to the manufacturer’s protocol.

All DNA was screened by an NGS custom panel, including genes/loci leading to the susceptibility to osteoporosis, as reported in the Online Mendelian Inheritance in Man (OMIM) database: *ALPL* (NM_000478), *COL1A1* (NM_000088), *COL1A2* (NM_000089), *CALCR* (NM_001164737.3), *LRP5* (NM_002335), *PDLIM4* (NM_003687.4), BMND7 (MIM 611738), BMND8 (MIM 611739).

In addition, *VDR* (NM_001017536), INSL3 (NM_005543.4), and RXFP2 (NM_130806.5) genes were included in the panel for their role on osteoblasts and osteocytes [37]. Furthermore, the panel included splice sites, 5′ untranslated regions (UTRs), and 3′ UTRs for each of the listed genes.

Probes were designed by the web-based application tool DesignStudio (Illumina Inc., San Diego, CA, USA) using the GRCh37/19 as the reference genome build. Probes created by DesignStudio were designed to cover 100% of the selected regions.

Sample libraries for sequencing were prepared starting from 100 ng of DNA by the Ampliseq Library kit according to the manufacturer’s protocol (Illumina, San Diego, CA, USA). Amplification libraries were assessed and quantified by the 4200 Agilent Tapestation System using the Agilent D1000 ScreenTape (Agilent, Santa Clara, CA, USA). Equimolar concentrations of libraries (2 nM) were combined, denatured, and diluted to a final concentration of 8 pM according to the manufacturer’s recommendation. The libraries were then loaded on a 500-cycle (2 × 250 paired ends) reagent cartridge (Illumina, San Diego, CA, USA), and run on a MiSeq sequencer (Illumina, San Diego, CA, USA).

For each run, the average depth was a minimum of ~ 100× coverage to allow for optimal variant calling. BWA-MEM and GATK were used as algorithms for aligning genome sequencing reads to the reference genome (GRCh37/hg19) and variant calling, respectively. Variants with an allelic frequency lower than 1% were confirmed by Sanger sequencing.

### 4.3. Variant Filtering and Interpretation of Clinical Relevance

Data were analyzed by VariantStudio software v.3.0 (Illumina, San Diego, CA, USA) and the Integrative Genomics Viewer (IGV) was used as a visualization tool for the detected variants. We used theavailable online dbSNP database, (https://www.ncbi.nlm.nih.gov/snp/) and Ensembl browser (http://grch37.ensembl.org/index.html) (accessed on 3 June 2021) to find the location and frequency of the variants.

Variant classification was performed following criteria of the American College of Medical Genetics and Genomics (ACMG) guidelines.

### 4.4. Bayesian Classificatory Model for LRP5 Pathogenicity Ranking

LRP5 mutations were ranked by developing a Bayesian classificatory model that integrates structural and protein features analysis in silico with data retrieved from the literature, as described in [25,26]. Multiple sequence alignment was calculated using T-coffe [38], while LRP5 ortholog protein sequences were retrieved by a search against the OMA browser database [39]. Domain organization was predicted using Pfam [40] and Uniprot [41], while secondary structure prediction and sequence feature identification were performed with Fells [42]. Previous pathogenic annotations were collected by a search against the ClinVar database [43]. The dbSNP database [44] was used to retrieve the frequency of each variant in the human population, while Polyphen2.0, SIFT, Pmut, and MuPro were used for pathogenicity and stability predictions [45,46,47,48]. Different lines of evidence were grouped in eight independent descriptors, i.e., sequence conservation, presence of functional domain, local sequence complexity, presence of structured domain, presence of functional linear motifs, agreement among in silico predictors, presence of previous pathogenic annotations in public databases, and presence of data in the literature. High risk was assigned to variants passing a minimum agreement for pathogenicity of 5/8 descriptors, numerically representing a baseline 62.5% odds ratio. Similarly, to avoid dangerous benignant misinterpretation, a neutral-like effect was limited to variants scoring below 2/8 descriptors (i.e., 25% baseline odds ratio).

### 4.5. Molecular Dynamics Simulation

LRP5 structural models were generated by automatic homology modeling prediction with the SWISS-Model [49] and selecting the best scoring structure as templates (accessed on May 2021). The resulting 3D-models of LRP5 repeated domains of both the wild-type and mutated protein were investigated for stability upon mutation by molecular dynamics simulations. A simulation environment was prepared using the CHARMM-GUI Input Generator [50], while calculations were carried out with GROMACS [51] using the CHARMM36 force field and the TIP3p explicit solvent model. A simulation run protocol consisted of 100 conjugate gradient minimization steps, 1000 ps under NVT conditions followed by 250 ns of classic molecular dynamics simulation at 310 K and 1.01325 bar. Integration was conducted using the Verlet method and imposing a 2 fs time step. The resulting trajectories were investigated in terms of RMSD and root-mean-square fluctuation (RMSF), while RING 2.0 [52] was used to estimate the variation in residue interaction network around mutated amino acid positions.

### 4.6. Statistical Analysis

Statistical analysis of the data was conducted with SPSS 21.0 for Windows (SPSS, Chicago, IL, USA). The results are expressed as means ± standard deviation. Student’s *t*-test was performed to compare the mean Z-score and T-score between groups.

## Figures and Tables

**Table 1:** Genetic variants and general characteristics of the carriers.

ID Patients	Protein, Codon	ACMG	Age	BMD_L_(g/cm^2^)	T_L_	Z_L_	BMD_TH(g/cm_^2^_)_	T _TH_	Z _TH_
11938	p.V99L, G>T	LP	55	0.90	−2.6	−2.3	0.86	−1.6	−0.9
9620	p.G333S, G>A	VUS	29	0.84	−2.2	−2.2	0.95	−0.5	−0.5
8957	p.E341K, G>A	VUS	28	0.67	−3.8	−3.8	0.78	−1.6	−1.6
12639	p.T443M, C>T	VUS	45	0.85	−2.2	−2.1	1.10	0.5	0.6
7971	p.R1036Q, G>A	LP	31	0.87	−2.0	−2.0	0.98	−0.3	−0.3
12141	p.R1135C, C>T	VUS	38	0.74	−3.1	−3.1	0.61	−2.7	−2.7
12025	p.R1342P, C>T	VUS	43	0.86	−2.0	−2.0	0.94	−0.6	−0.5
11241	p.A1525V, C>T	B	43	0.86	−2.1	−2.0	0.81	−1.4	−1.2
3801	p.A1525V, C>T	B	34	0.81	−2.5	−2.5	0.91	−0.8	−0.7
10517	p.A1537V, C>T	VUS	48	0.81	−2.5	−2.4	1.04	0.0	0.3
12,007	p.S1585L, C>T	VUS	36	0.71	−3.4	−3.4	0.75	−1.8	−1.7

ACMG: American College of Medical Genetics and Genomics; BMD, bone mineral density; ZL, Z score at the lumbar spine; TL, T score at the lumbar spine; ZTH, Z score at the total hip; TH, T score at the total hip. LP: likely pathogenic; VUS: variant unknown significance; B: benign.

**Table 2:** Pathogenicity and stability predictions of LRP5 variants.

Variant	PolyPhen-2	SIFT	Pmut	MuPro ΔΔG
p.V99L	Benign	Tolerated	Neutral	−0.44734934 (DECREASE stability)
p.G333S	Benign	Tolerated	Neutral	−1.3066037 (DECREASE stability)
p.E341K	Benign	Tolerated	Disease	−0.82809476 (DECREASE stability)
p.T443M	Probably damaging	Tolerated	Neutral	−0.26659723 (DECREASE stability)
p.R1036Q	Possibly damaging	Tolerated	Neutral	−1.171288 (DECREASE stability)
p.R1135C	Probably damaging	Tolerated	Disease	−0.47636602 (DECREASE stability)
p.R1342P	Probably damaging	Tolerated	Disease	−0.94002976 (DECREASE stability)
p.A1525V	Benign	Tolerated	Neutral	−0.35033282 (DECREASE stability)
p.A1537V	Benign	Not tolerated	Neutral	−0.31014871 (DECREASE stability)
p.S1585L	Probably damaging	Not tolerated	Disease	−0.30474209 (DECREASE stability)

**Table 3:** Classification of LRP5 variants using a Bayesian integrated analysis.

Variant	Prediction Score	Odd Score	Merged Score In Silico	Prior OR	Posterior OR	Posterior OR%	Meaning	Class
p.V99L	1	1	2	1	2	22.22	LN	2
p.G333S	1	1	2	1	2	22.22	LN	2
p.E341K	2	3	5	1	5	55.56	LP	4
p.T443M	2	3	5	1	5	55.56	LP	4
p.R1036Q	2	5	7	1.25	8.745	97.22	P	5
p.R1135C	2	5	8	1.25	10	111.11	P	5
p.R1342P	3	3	6	1	6	66.67	LP	4
p.A1515V	2	3	5	1.25	6.25	69.44	LP	4
p.A1537V	2	3	5	1	5	55.56	LP	4
p.S1585L	4	1	5	1	5	55.56	LP	4

LN = Likely Neutral; LP = Likely Pathogenic; P = Pathogenic.

**Table 4:** General characteristics (mean ± SD) of men with altered bone mass.

	Patient (*N* = 128)
Age (years)	41.6 ± 8.7
Height (cm)	178.1 ± 5.6
Weight (kg)	77.8 ± 8.4
BMI (kg/m^2^)	23.1 ± 2.2
BMD_L_ (g/cm^2^)	0.82 ± 0.07
T_L_	<−2.5 ± 0.55
Z_L_	<−2.48 ± 0.59
BMD_L_ (g/cm^2^)	0.88 ± 0.11
T_TH_	<−1.07 ± 0.79
Z_TH_	<−0.99 ± 0.72

BMI: Body Mass Index; BMD: Bone Mineral Density; TL, T score at the lumbar spine; ZL, Z score at the lumbar spine; TTH, Z score at the total hip region; ZTH, Z score at the total hip region.

## Data Availability

The raw sequencing data are available in NCBI’s BioProject under the accession number SUB9808924, BioProject: PRJNA735686 (https://dataview.ncbi.nlm.nih.gov/?search=SUB9808924&archive=bioproject).

## Supplementary Materials

The following are available online at https://www.mdpi.com/article/10.3390/ijms221910834/s1
