# Supplementary figures and images for: Identification of Rare LRP5 Variants in a Cohort of Males with Impaired Bone Mass
Source: Int J Mol Sci. 2021 Oct 7;22(19):10834. doi: 10.3390/ijms221910834 (PMC8509722; doi:10.3390/ijms221910834)

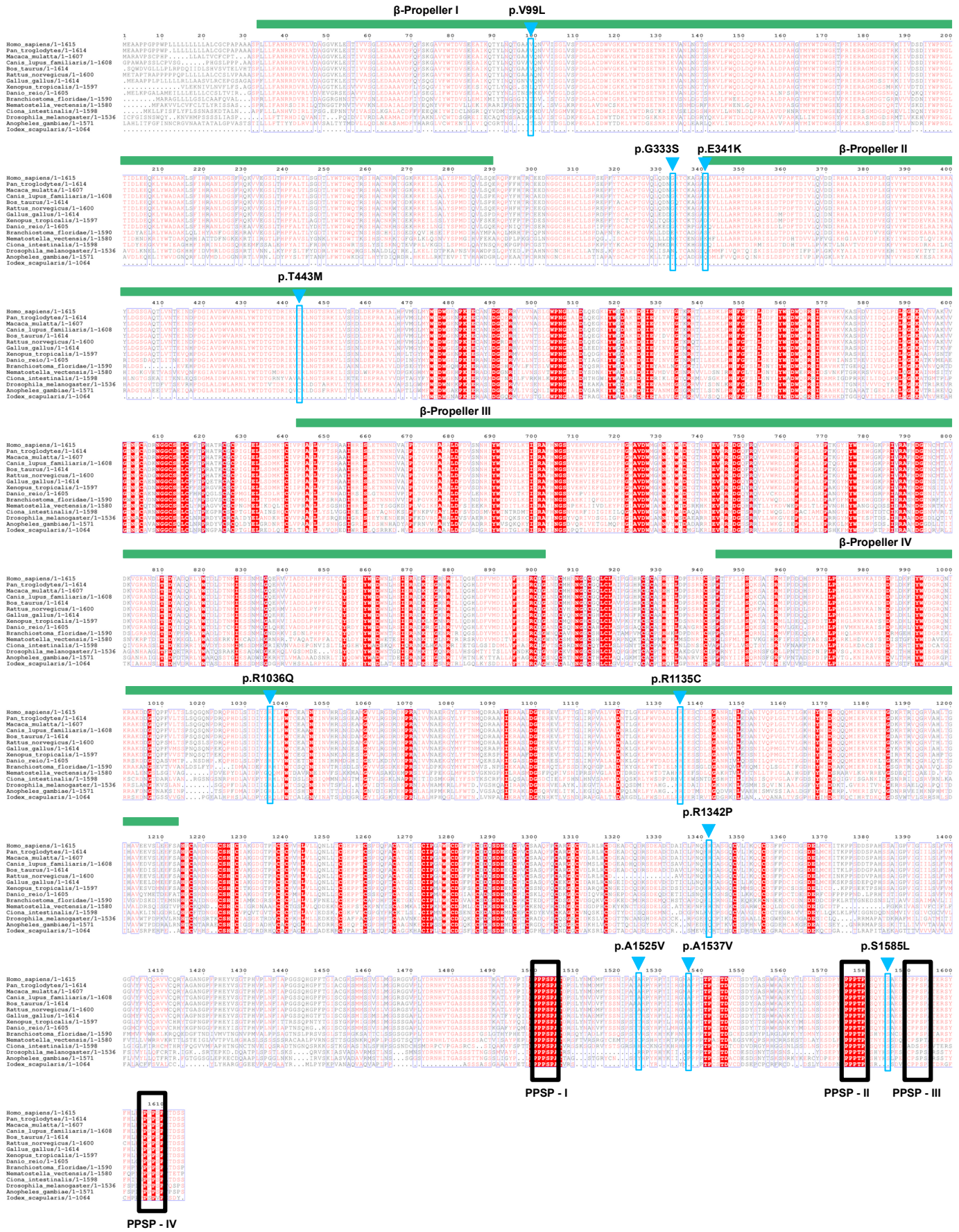

Supplement: Supplementary file 1 [file ijms-22-10834-s001.zip › Supplementary_Materials/Supplementary_Figure_S1.pdf]
